# Exosomes derived from 5-fluorouracil-resistant colon cancer cells are enriched in GDF15 and can promote angiogenesis

**DOI:** 10.7150/jca.49224

**Published:** 2020-10-18

**Authors:** Xixi Zheng, Nina Ma, Xingyu Wang, Jiexuan Hu, Xiao Ma, Jingting Wang, Bangwei Cao

**Affiliations:** 1 Department of Oncology, Beijing Friendship Hospital, Capital Medical University, Beijing, 100050, China.; 2 Department of Gastroenterology, National Clinical Research Center for Digestive Disease, Beijing Digestive Disease Center, Beijing, 100050, China.

**Keywords:** Colon cancer, 5-FU resistance, Exosomes, GDF15, Angiogenesis

## Abstract

**Background:** Angiogenesis is important for tumor proliferation and distant metastasis. However, the role of drug-resistant tumor cells in angiogenesis remains largely unknown. Current anti-angiogenic strategies also have limitations and it would be useful to develop novel targets and treatment strategies.

**Methods:** Differential ultracentrifugation was used to isolate conditioned medium-derived exosomes from 5-flurouracil (5-FU)-sensitive or -resistant colon cancer cells. Exosome endocytosis into human umbilical vein endothelial cells was observed via immunofluorescence. Differentially expressed proteins in the exosomes were confirmed via qRT-PCR and Western blotting. The angiogenic capacity of endothelial cells was evaluated using cell function assays and a rat model of abdominal aortic neovascularization. The underlying mechanisms were verified using qRT-PCR and Western blotting assays. Immunohistochemistry was used to evaluate in vivo angiogenesis.

**Results:** We observed that the conditioned medium and exosomes from 5-FU-resistant colon cancer cells could promote angiogenesis. Exosomal growth/differentiation factor 15 (GDF15) was a potent inducer of this angiogenesis in vitro by inhibiting the Smad signaling pathway, thus increasing periostin (POSTN) levels. Moreover, 5-FU-resistant colon cancer cells showed high microvascular density in vivo. TGF-β1, an activator of the Smad signaling pathway, could partly eliminate those effects.

**Conclusions:** Our study reveals the molecular regulation of angiogenesis in 5-FU-resistant colon cancer and suggests that the GDF15-POSTN axis may be a novel target for anti-angiogenic therapies in colon cancer.

## Introduction

Colon cancer is one of the most common malignancies 1. Chemotherapy based on 5-fluorouracil (5-FU) is the standard approach to the treatment of colon cancer. Resistance to 5-FU is the primary cause of disease progression in colon cancer patients. Tumor cells are known to produce a variety of compounds to mediate their microenvironment. Drug-resistant cells exhibit accelerated proliferation and distant metastasis, along with increased angiogenesis 2. Studies into the mechanism of angiogenesis in drug-resistant tumor cells in the microenvironment are therefore urgently needed.

Exosomes are small (30-200 nm) lipid bilayer membrane vesicles, which are secreted by many cells, both normal and cancerous, into the cell supernatant. These extracellular vesicles contain RNA, proteins or bioactive lipids, and can transfer these biomolecules between cells, thus playing an essential role in the development and progression of tumors 3. Evidence indicates that tumor-derived exosomes can enhance tumor growth, drug resistance and metastasis in a paracrine or autocrine fashion, which can contribute to cancer pathogenesis 2, 4-6. However, few studies have focused on the impact of exosomes on the angiogenesis of tumors, especially in colon cancer.

Many proangiogenic and antiangiogenic molecules affect tumor angiogenesis. Among these, the vascular endothelial growth factor (VEGF) plays a crucial role in facilitating angiogenesis, and has significant potential as an angiogenic target 7, 8. Despite early clinical benefits obtained using anti-angiogenesis therapy targeting VEGF, most patients eventually experience tumor recurrence or distant metastasis. Therefore, it is critical to identify additional factors and pathways independent of VEGF signaling, which could add to the arsenal of anti-angiogenesis therapies for colon cancer patients.

Growth/differentiation factor 15 (GDF15), a member of the TGF-β/bone morphogenetic protein (BMP) super-family, is expressed in many types of tissues and has become the subject of considerable attention in research into cancer progression 9, 10. However, the role of GDF15 in tumor development is not well understood, and some of the data which have been obtained are contradictory 11, 12. GDF15 can be actively secreted into the microenvironment by a variety of cells. As previously reported, secreted GDF-15 could regulate the tumor microenvironment and facilitate the proliferation, invasion, and metastasis of tumors by affecting the activity of downstream signaling pathways 10, 13-15. The role of secreted GDF-15, especially with respect to drug resistance and angiogenesis, is not yet fully understood.

Therefore, we investigated the relationship between drug resistance in cancer cells and angiogenesis. Further, we determined the role of GDF-15 secreted by exosomes from cancer cells in angiogenesis and its underlying mechanism. Our findings will provide new insights into the potential of GDF15 as a novel anti-angiogenic target for colon cancer therapy.

## Materials and Methods

### Cell culture and conditioned medium

HCT-15 (human colon cancer cells) and HCT-15/FU cells were bought from the cell bank of Chinese Academy of Sciences (Shanghai, China) and were cultured in RPMI 1640 medium containing 10% fetal bovine serum (FBS, Gibco, USA). HCT-15/FU cells were supplemented with 3.2 μg/ml 5-FU. Conditioned medium (CM) was collected after cells were cultured in exosome-free FBS medium for 48 h. Human umbilical vein endothelial cells (HUVECs) were purchased from the cell bank of Chinese Academy of Sciences (Shanghai, China) and were cultured in endothelial cell medium (ECM) obtained from ScienCell Research Laboratories. Before treatment of exosomes, cells were cultured in basal medium. All cells were incubated at 37°C with 5% CO_2_.

### Exosomes isolation and labeling

Exosomes were isolated from HCT-15 and HCT-15/FU cells conditioned medium by differential centrifugation. CM was centrifuged at 2 500 ×g and 8 500 ×g for 30 min each time. Then, the supernatant was centrifuged at 120 000 ×g for 70 min three times. Exosomes were further centrifuged at 110 000 ×g for 70 min (all steps were performed at 4 °C) and re-suspended in PBS. The concentration of exosomes was determined using the BCA method (ThermoScientific, USA). Exosomes were labelled as recommended by the manufacturer (PKH67 kit, Sigma Aldrich).

### Transmission electron microscopy assay

Exosomes were first diluted in PBS and then placed on copper grids. The grids were stained with 1% (v/v) uranyl acetate in ddH_2_O after a minute. Sample morphology was detected by transmission electron microscopy (TEM, Hitachi, Japan).

### Live-cell imaging assay

In total, 3 × 10^3^ HUVECs were cultured in each well of a 96-well plate and treated with CM or exosomes with or without TGF-β1. Phase object confluence was measured with the extension of time using IncuCyte S3.

### Cell proliferation assay

HUVECs were incubated with 50 mM EdU (RiboBioInc) for 2 h and fixed with 4% paraformaldehyde for 60 min. Cells were permeabilized using 0.5% Triton X-100 for 10 min and then stained with Apollo (RiboBio, Inc.) for 40 min and Hoechst33342 (1:100; RiboBio Inc.) for 30 min. Cells were washed gently with PBS between each step.

### Cell migration assay

The migration ability of HUVECs was tested in a Transwell Boyden Chamber (8 mm pore size, 6.5 mm diameter) seeded in the upper chamber with 2 × 10^4^ HUVECs in 0.5 ml CM or exosomes with or without TGF-β1, the lower chamber was filled with 0.8 ml serum-free medium. After incubating for 48 h, the lower chamber was replaced with medium containing 10% exosome-free FBS and incubated for another 10-12 h. Then, cells were fixed with 100% methanol for 20 min and stained with 0.1% crystal violet for 15 min. The cells in the upper compartment were wiped off. Images were taken using a photo-microscope and quantified by counting at least three fields.

### Wound-healing assay

HUVECs were cultured in 24-well plates with CM or exosomes with or without TGF-β1. At 100% confluency, a 10-µL pipette tip was used to scratch a wound at the center of cell monolayer in the culture plates. The wounds between cells were washed twice with PBS and culture medium replaced with the same fresh medium for another 48 h. Images of wounds were captured at 0, 12, 24, 36 and 48 h, and the wound healing area was calculated by ImageJ software.

### Tube formation assay

HUVECs were treated with CM or exosomes with or without TGF-β1 for at least 48 h at 37 °C and redistributed at a density of 1 × 10^4^ in 96-well plates with 60 ul Matrigel per well. Further incubation at 37 °C was performed for 6-8 h. Images of at least three fields per well were captured by phase contrast microscopy (×10). ImageJ software was used to count and analyze the number of branch points of the tubes per field.

### Rat abdominal aortic neovascularization assay

Sprague-Dawley rats (6 weeks old) were anaesthetized with 10% chloral hydrate. Left ventricular perfusion was used to remove as much blood as possible from the arteries. The abdominal aorta was isolated and washed with PBS, and cut into 1-2 mm vascular rings. The ring was placed in a 96-well plate with 70 μl Matrigel in the bottom, and additional 70 μl Matrigel was added on the ring to form a sandwich structure. After incubated at 37°C for one hour, the rings were treated with CM or exosomes with or without TGF-β1. The medium was changed every two days. Images were taken by inverse microscope, and the area of neovascularization was counted and analyzed by ImageJ software.

### Immunofluorescence assays

In total, 1.5 × 10^5^ HUVECs were seeded on slides in a 6-well plate and cultured to 50% fusion density. Then, 500 μl PKH67 labeled exosomes were added to the plate and cells were co-cultured for 8-10 h. The cells were fixed with 4% PFA and cell membrane was ruptured with 0.3% Triton X-100 for 15 min each. The cells were blocked with 5% fetal bovine serum albumin for 45 min, and incubated with the primary antibodies (ZO-1, Abcam) at 4°C overnight and the fluorescent secondary antibody (Alexa Fluor 568 goat anti-rabbit IgG, Life Technologies) at room temperature for 2 h. Cells were stained with DAPI. Images were captured using confocal microscopy (IX83, FLUOVIEW FV1200; Olympus).

### RNA extraction and quantitative reverse transcriptase-PCR (qRT-PCR)

Total RNA was extracted from cultured cells using TRIzol Reagent (Invitrogen) according to the manufacturer's instructions. cDNA was obtained from RNA using a reverse transcriptase kit (Takara, China), and quantitative real-time PCR was performed with SYBR Premix ExTaqTM II (Takara, Dalian, China). The cycling conditions were 25˚C for 10 min, 37˚C for 2 min, followed by 85˚C for 5 min. The mRNA levels were normalized to GAPDH and 2^-ΔΔCt^ was used for data statistics. The primers used in qRT-PCR are listed in Table S1.

### Western blotting assay

Whole proteins were extracted from cells or exosomes using immunoprecipitation buffer and quantified using BCA protein assay kit (Thermo scientific Pierce). After separation on a 10% SDS-PAGE gel, proteins were transferred to a PVDF membrane (Millipore) and blocked in 5% nonfat milk. Proteins were incubated with primary antibodies overnight at 4°C and secondary antibodies at room temperature for 2 h. TBST buffer was used to wash off the unbound antibodies. Protein bands were visualized by an ECL plus system (Beyotime). The specific primary antibodies used in western blot analysis were as follows: anti-CD9 (1:2000, Abcam), anti-CD63 (1:1000, Abcam), anti-TSG101 (1:1000, Abcam), anti-GM130 (1:2000, Proteintech), anti-GDF15 (1:1000, Abcam), anti-p-FAK (1:500, Immunoway), anti-FAK (1:1000, Immunoway), anti-AKT (1:1000, Abcam), anti-p-AKT (1:1000, CellSignaling Technology), anti-POSTN (1:500, Hangzhou HuaAn Biotechnology), anti-TGBR3 (1:500, Immunoway), anti-p-Smad2/3 (1:500, Hangzhou HuaAn Biotechnology), anti-Smad2/3 (1:1000, Hangzhou HuaAn Biotechnology), anti-GAPDH (1:5000, Proteintech).

### Establishment of tumor in nude mice

Sixteen male BALB/c-nu mice (4-6 weeks old) were randomly divided into four groups. The mice were inoculated subcutaneously with HCT-15 or HCT-15/FU cells (1 × 10^7^ cells in 100 μL PBS, respectively) in the groin. One week later, 1.27mM 5-FU was injected into developed tumor every other day. In addition, HCT-15/FU groups were also treated with citrate or TGF-β1. After 28 days, all the mice were sacrificed by cervical dislocation, and the tumors were excised, measured, weighed, and photographed. All animal experiments were performed in accordance with a protocol approved by the experimental animal welfare ethics review committee of National Institutes for Food and Drug Control, China.

### Immunohistochemistry (IHC)

Paraffin sections were deparaffinized and hydrated, and then rinsed three times with PBS, each time for 3 minutes. Next, the tissues were subjected to high-pressure antigen repair (Tris-EDTA PH9.0) for 2.5 min, treated with 3% endogenous catalase blocker (ZSBIO) for 15 min, and blocked using goat serum (ZSBIO). This was followed by incubation of the tissues with primary antibody at 37°C for 2-3 h and secondary antibody (ZSBIO) for one hour at room temperature. Tissues were washed with PBS and stained with DAB reagents (ZSBIO). Finally, the tissues were stained hematoxylin, differentiated with 1% hydrochloric acid alcohol, and sealed using neutral gum sealed. Results were evaluated by pathologists. The specific primary antibody used in IHC is anti-CD34 (1:1000, ZSBIO).

### Statistical analysis

Statistical analysis was conducted using GraphPad Prism 5.0 software. Data were expressed as mean ± SEM. The differences between two groups were analyzed by student's t-test, and three groups or more were by one-way ANOVA.* P*< .05 was considered statistically significant.

## Results

### Conditioned medium (CM) derived from 5-FU-resistant colon cancer cells promotes angiogenesis

**Figure 1:**
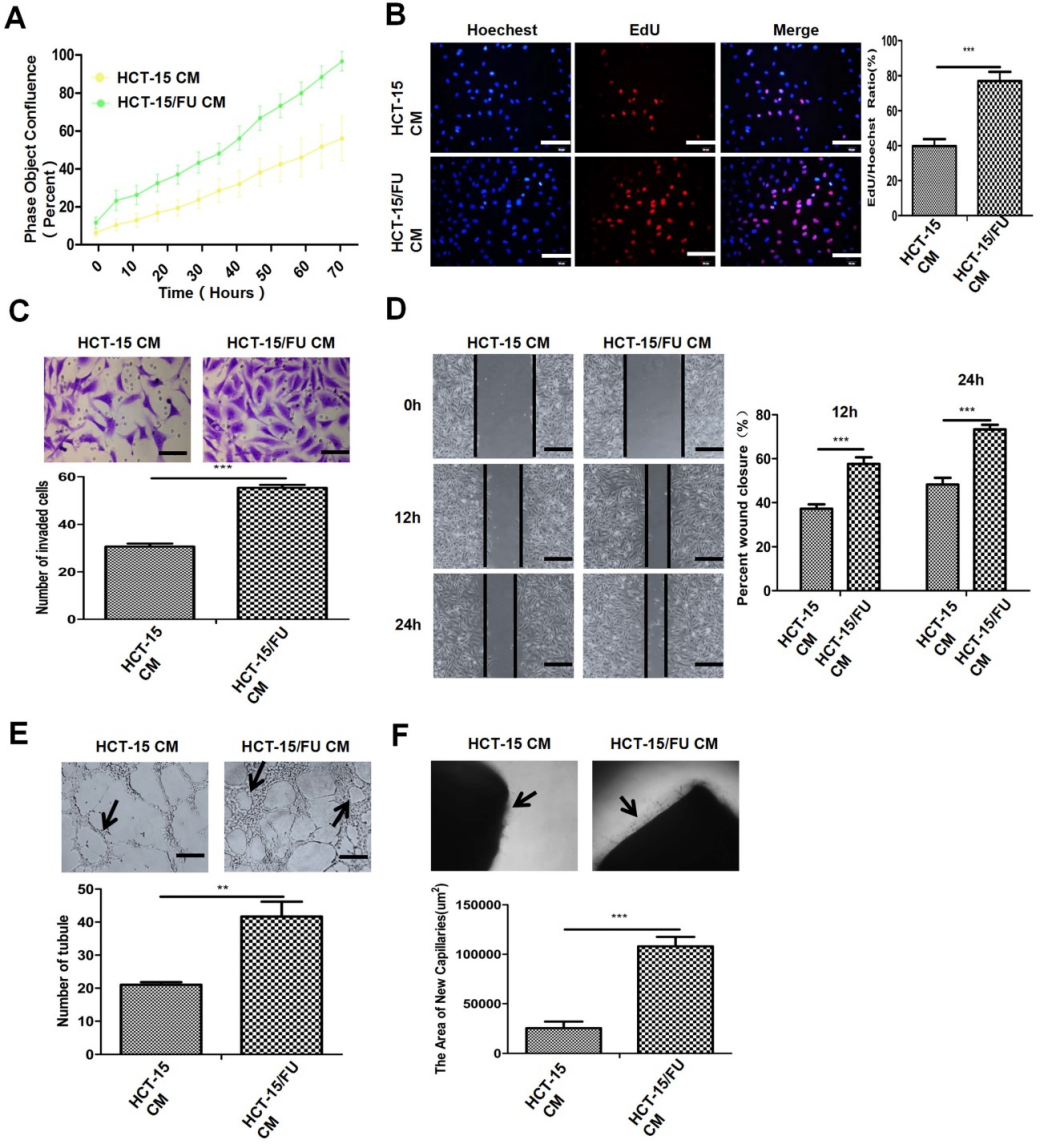
** Conditioned medium (CM) derived from 5-FU-resistant colon cancer cells promotes angiogenesis. A** and **B.** Proliferation assay of HUVECs treated with CM of HCT-15 and HCT-15/FU cells. **(A)** Live-cell imaging experiment and **(B)** EdU experiment, Scale bars, 100 μm. **C** and **D.** Cell migration assay of HUVECs treated with CM from HCT-15 or HCT-15/FU cells.** (C)** Transwell migration assay, Scale bars, 50 μm and **(D)** Wound-healing assay, Scale bars, 200 μm. **E.** Tube formation assay of HUVECs treated with CM of HCT-15 or HCT-15/FU cells. Scale bars, 200 μm. **F.** Rat abdominal aortic neovascularization assay. Scale bars, 200 μm. The data represent the mean ± SEM. Statistical significance *P< .05, **P< .01 and ***P< .001.

Tumor angiogenesis is a multi-step process, in which endothelial cells have an increased proliferation rate, migrate through the extracellular matrix, and then form new blood vessels. We investigated these processes in order to study the mechanisms of angiogenesis. We used 5-FU-sensitive colon cancer HCT-15 cells and resistant colon cancer HCT-15/FU cells (IC_50_ were 1.27 mM and 10.38 mM, respectively. Figure S1). We generated the HCT-15/FU cells from parental HCT-15 cells by continuous exposure to gradually increasing concentrations of 5-FU. We then collected the CM of both cells and co-cultured them with human umbilical vein endothelial cells (HUVECs). We found that the CM of HCT-15/FU cells could increase the proliferation (Figure 1A, B), migration (Figure 1C, D) and tube formation (Figure 1E) of HUVECs, compared with that of HCT-15 cells. To further investigate the effects of CM on angiogenesis, we employed a rat model of abdominal aortic neovascularization. As shown in Figure 1F, the CM of HCT-15/FU cells caused an increase in the number of microvessels surrounding the rat abdominal aorta. Collectively, our results suggest that the CM of 5-FU-resistant HCT-15/FU cells can enhance the proliferation, migration, and tube formation ability of HUVECs, as well as the density of microvessels surrounding the aorta.

### HCT-15/FU cell-derived exosomes accelerate angiogenesis

**Figure 2:**
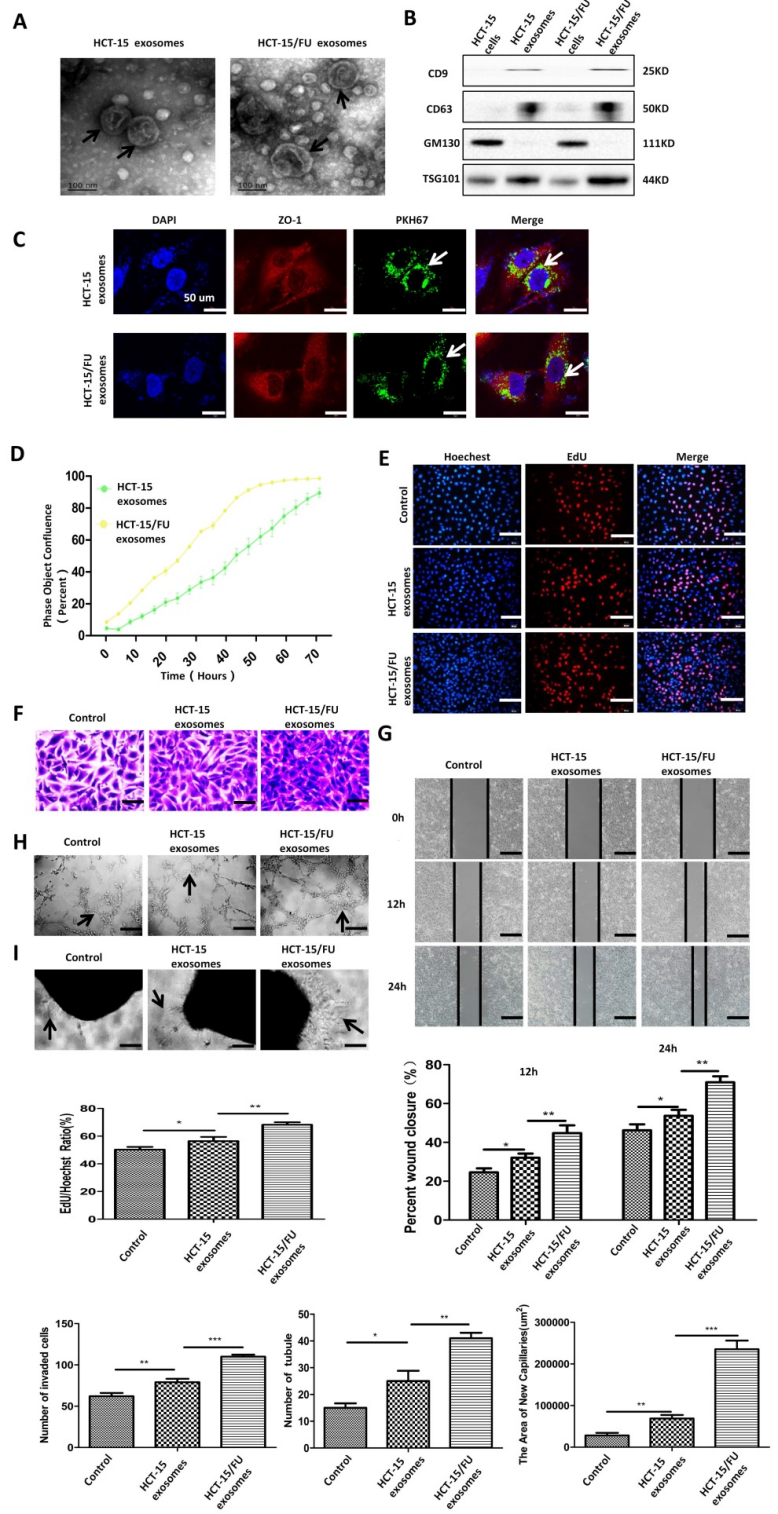
** HCT-15/FU cell-derived exosomes accelerate angiogenesis. A.** Transmission electron microscopy (TEM) images of exosomes from HCT-15 and HCT-15/FU cells. **B.** Western blottinig analyses of exosomal positive (CD63, CD9 and TSG101) and negative (GM130) markers. **C.** Confocal microscopy showed the internalization of exosomes. **D and E.** Proliferation assay of HUVECs treated with HCT-15 or HCT-15/FU exosomes. **(D)** Live-cell imaging experiment and **(E)** EdU experiment, Scale bars, 100 μm. **F** and **G.** Cell migration assay of HUVECs treated with HCT-15 or HCT-15/FU exosomes. **(F)** Transwell migration assay, Scale bars, 50 μm and **(G)** Wound-healing assay, Scale bars, 200 μm. **H.** Tube formation assay of HUVECs treated with HCT-15 or HCT-15/FU exosomes. Scale bars, 200 μm. **I.** Rat abdominal aortic neovascularization assay. Scale bars, 200 μm. The data represent the mean ± SEM. Statistical significance *P <0 .05, **P <0.01 and ***P <0.001.

Based on previous research, and considering the increasing importance of exosomes in cellular information transmission, we suspected that exosomes might be involved in the promotion of angiogenesis by CM. We extracted exosomes from HCT-15 and HCT-15/FU cells by performing differential ultracentrifugation, and confirmed their presence using transmission electron microscopy (TEM). The exosomes appeared as double-layered disc structures with an average diameter of 100-120 nm (Figure 2A). Western blotting was used to verify the level of the exosomal positive markers CD9, CD63, and TSG101, and the negative marker GM130 in these vesicles, further confirming that the structures isolated were exosomes (Figure 2B). The exosomes were labeled with PKH67 and incubated with HUVECs. HUVECs were observed to internalize the green fluorescent protein-tagged exosomes after 10 hours (Figure 2C).

We then performed a live-cell imaging experiment to observe the effect of the exosomes on HUVEC proliferation. Compared with HCT-15 exosomes, HCT-15/FU exosomes increased the proliferation of HUVECs, especially after incubation with 40 µg/ml for 48 h. (Figure 2D, Figure S2). The results of the EdU experiment showed a similar trend (Figure 2E). Based on these results, a concentration of 40 μg/mL of exosomes and an incubation time of 48 h were used in the following experiments. The exosomes consistently enhanced endothelial cell migration (Figure 2F, G), tube formation (Figure 2H). Increased abdominal aortic neovascularization (Figure 2I) was also observed after co-culture with HCT-15/FU exosomes. These results suggest that HCT-15/FU exosome could enhance angiogenesis in vitro.

### HCT-15/FU exosomes are enriched in GDF15 and improve angiogenesis via inhibition of the Smad signaling pathway

**Figure 3:**
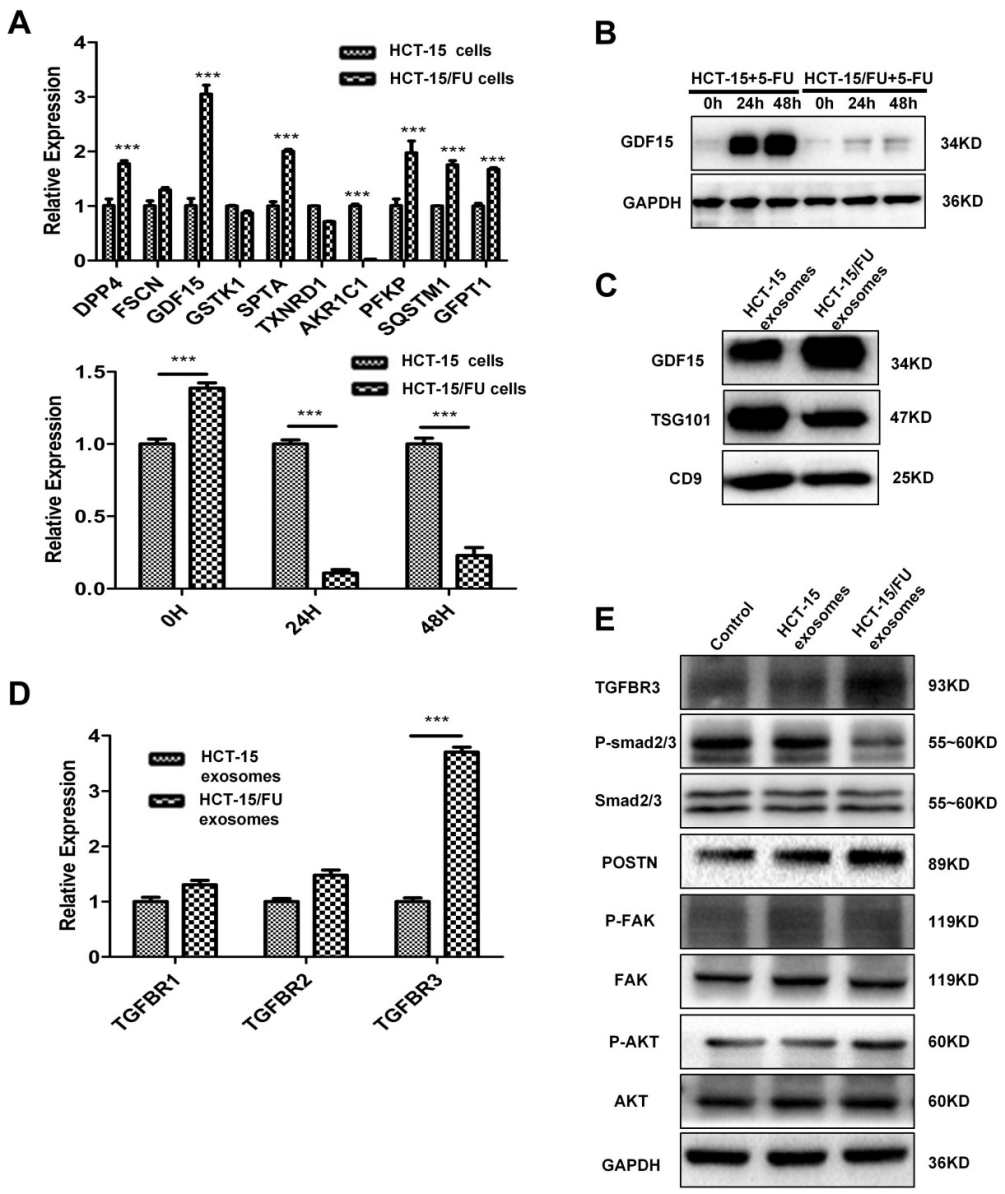
** HCT-15/FU exosomes are enriched in GDF15 and improve angiogenesis via inhibition of the Smad signaling pathway. A.** Upper: Relative mRNA expression of the top ten differentially expressed proteins in HCT-15 and HCT-15/FU cells. Lower: mRNA level of GDF15 in HCT-15 and HCT-15/FU cells treated with 5-FU. **B and C.** Western blotting analysis of GDF15 levels in HCT-15 and HCT-15/FU cells and exosomes. **(B)** GDF15 level in HCT-15 and HCT-15/FU cells and** (C)** GDF15 level in HCT-15 and HCT-15/FU exosomes. **D.** Relative mRNA expression of TGF-β receptors in HUVECs co-cultured with HCT-15 or HCT-15/FU exosomes.** E.** The expression of TGBR3, p-Smad2/3 ,Smad2/3, POSTN and downstream pathway moleculars p-FAK, FAK, p-AKT, AKT in HUVECs treated with HCT-15 or HCT-15/FU exosomes using western blot. The data represent the mean ± SEM. Statistical significance ***P < 0.001.

HCT-15/FU exosomes are clearly important in promoting angiogenesis, but the underlying mechanisms are not fully understood. On the basis of our previous work using label-free quantitative analysis of exosomes from another pair of 5-FU-resistant and -sensitive colon cancer cell lines, HCT-8 and HCT-8/FU, we examined the mRNA levels of the 10 most abundant proteins in HCT-15 and HCT-15/FU cells, which were elevated in HCT-8/FU exosomes. Among them, the expression of GDF15 in HCT-15/FU cells was higher than that in HCT-15 cells by approximately 3.1 fold (Figure 3A). With increasing 5-FU treatment time, both the mRNA and protein levels of GDF15 decreased in HCT-15/FU cells compared with HCT-15 cells (Figure 3A, B). We investigated the expression of GDF15 in exosomes from HCT-15 and HCT-15/FU cells. As shown in Figure 3C, the GDF15 level in HCT-15 /FU exosomes was higher than in HCT-15. Based on previous studies into the intracellular function of GDF15, we hypothesized that chemotherapy caused drug-resistant cells to secrete the proteins to the extracellular matrix. The secreted GDF-15 could then act as a tumor promoter. Since HCT-15/FU exosomes promote angiogenesis, we were able to demonstrate that GDF15 was associated with 5-FU resistance in HCT-15/FU cells. GDF15 was discharged into exosomes by drug-resistant cells after 5-FU chemotherapy, which subsequently regulated the tumor microenvironment through mechanisms such as angiogenesis.

GDF15 is a member of the TGF-β/bone morphogenetic protein (BMP) super-family. Like other members, GDF15 affects a variety of biological functions by regulating the TGF-β/Smad signaling pathway. We explored the mechanism by which GDF15 mediates the TGFβ pathway. We identified TGFBR3 as a potential target by measuring its mRNA levels in HUVECs co-cultured with HCT-15 or HCT-15/FU exosomes (Figure 3D). Activation of this receptor inhibits TGFβ1 signaling 16. Western blotting was used to verify the inhibition of TGFβ1 downstream signaling, as evidenced by a decrease in phospho-Smad2/3 in HUVECs treated with HCT-15/FU exosomes (Figure 3E). Smad is known to affect the transcription of POSTN, and POSTN plays a role in promoting angiogenesis 17-19. We measured the levels of POSTN in HUVECs after treatment with HCT-15 or HCT-15/FU exosomes. The presence of HCT-15/FU exosomes increased the expression of POSTN and subsequently increased levels of phospho-AKT and phospho-FAK, downstream effectors of POSTN in HUVECs, compared to those exposed to HCT-15 exosomes (Figure 3E). These results support that HCT-15/FU exosomes contain high levels of GDF15, which can inhibit the Smad signaling pathway to increase POSTN expression and promote endothelial cell angiogenesis.

### TGFβ1 can neutralize the increased angiogenesis caused by HCT-15/FU exosomes

**Figure 4:**
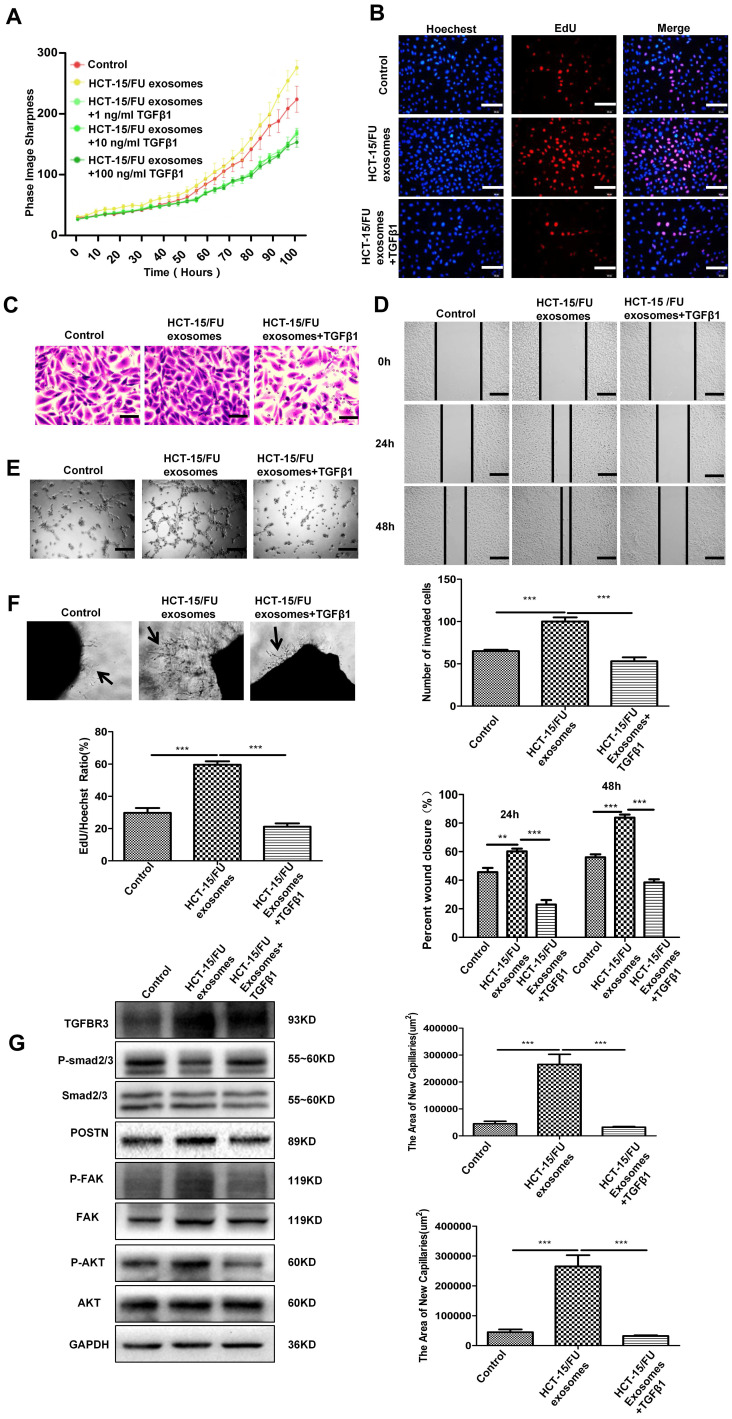
** TGFβ1 can neutralize the increased angiogenesis caused by HCT-15/FU exosomes. A and B.** Proliferation assay of HUVECs treated with control (citrate) or HCT-15/FU exosomes (40 μg/mL) with or without TGFβ1. **(A)** Live-cell imaging experiment (TGFβ1: 1, 10, 100 ng/ml) and **(B)** EdU experiment (TGFβ1, 1 ng/ml). Scale bars, 100 μm. **C and D.** Cell migration assay of HUVECs treated with control (citrate) or HCT-15/FU exosomes (40 μg/mL) with or without 1 ng/ml TGFβ1. **(C)** Transwell migration assay, Scale bars, 50 μm and **(D)** Wound-healing assay, Scale bars, 200 μm. **E.** Tube formation assay of HUVECs treated with control (citrate) or HCT-15/FU exosomes (40 μg/mL) with or without 1 ng/ml TGFβ1. Scale bars, 200 μm. **F.** Rat abdominal aortic neovascularization assay. The abdominal aorta rings were embedded in Matrigel and incubated in control (citrate) or HCT-15/FU exosomes (40 μg/mL) with or without 1 ng/ml TGFβ1. Scale bars, 200 μm. **G.** Western blotting analysis of TGBR3, p-Smad2/3, Smad2/3, POSTN and downstream pathway moleculars p-FAK, FAK, p-AKT, AKT in HUVECs treated with control (citrate) or HCT-15/FU exosomes (40 μg/mL) with or without 1 ng/ml TGFβ1 (n=3). The data represent the mean ± SEM. Statistical significance *P <0 .05, **P < 0.01 and ***P < 0.001.

Previous studies have reduced the expression of related proteins in exosomes by knocking down in cells with siRNA 2. In this study, we found that the GDF15 was highly expressed in HCT-15/FU exosomes, but expressed at low levels in HCT-15/FU cells. Exosomal GDF15 can promote endothelial cell angiogenesis via inhibiting the Smad signaling pathway. We next investigated the effect of TGFβ1, an activator of the TGF-β/Smad signaling pathway, on angiogenesis to confirm our findings further. HUVECs were incubated with 40 ug/ml HCT-15/FU exosomes with or without gradually increased concentrations of TGFβ1. As shown in Figure 4A, 1 ng/ml TGFβ1 significantly weakened the effect of HCT-15/FU exosomes on the proliferation of HUVECs. This inhibitory effect remained unchanged even with increasing concentrations of TGFβ1. Therefore, 1 ng/ml TGFβ1 was used in the subsequent experiments. EdU assay identified a similar weakening effect on proliferation (Figure 4B). TGFβ1 also blocked the enhanced endothelial cell migration (Figure 4C, D), tube formation (Figure 4E) and the increase in numbers of microvessels surrounding the rat abdominal aorta (Figure 4F). We studied changes in the relevant signaling pathway proteins using western blotting. The downregulation of phospho-Smad2/3 and upregulation of TGBR3, POSTN, phospho-FAK, and phospho-AKT in HUVECs stimulated by HCT-15/FU exosomes were reversed (Figure 4G). These results indicated that activation of the TGFβ1 signaling pathway could, at least in part, abolish the increased tubular capacity of endothelial cells induced by GDF15-enriched HCT-15/FU exosomes.

### HCT-15/FU cells show high microvascular density in vivo and can be weakened by TGFβ1

**Figure 5:**
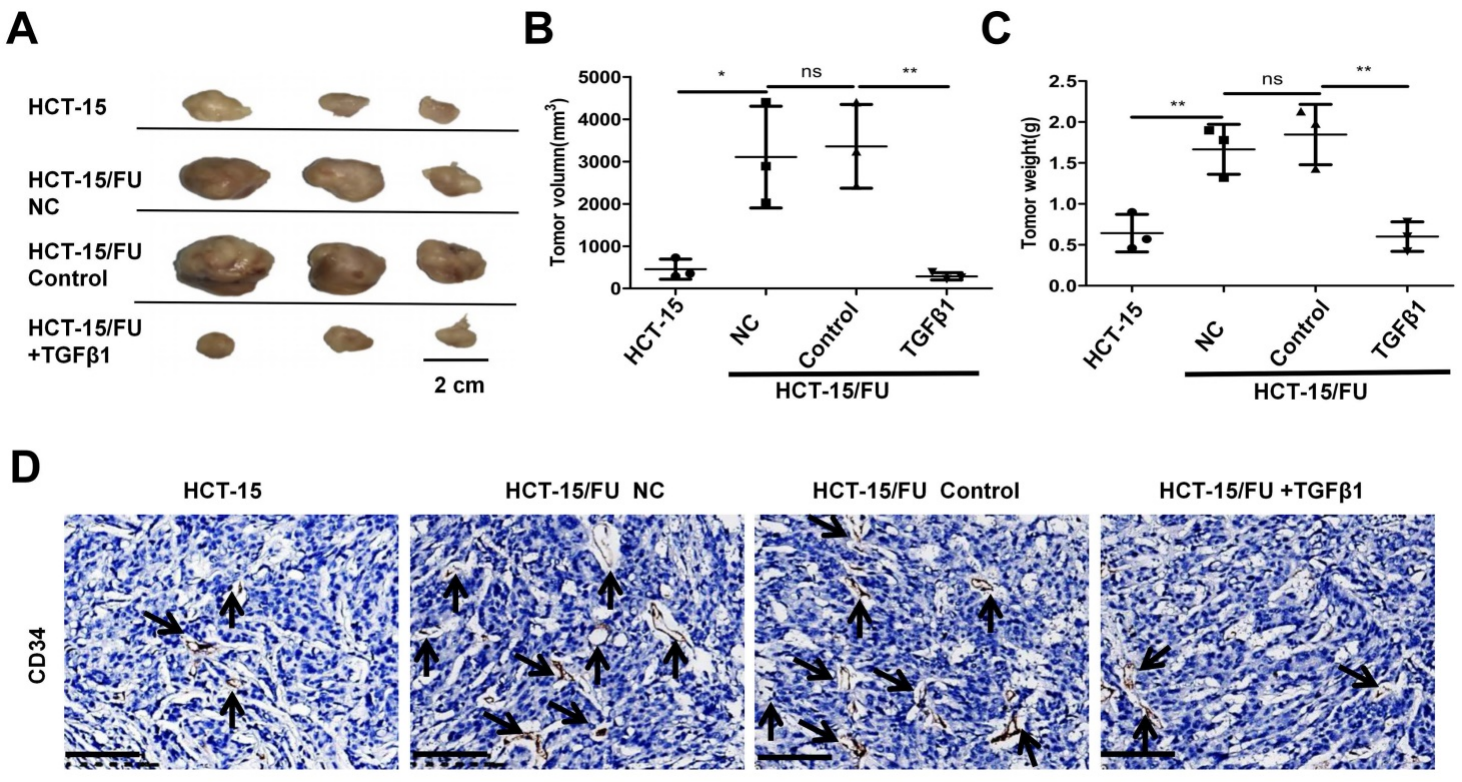
** HCT-15/FU cells show high microvascular density in vivo and can be weakened by TGFβ1. A.** The appearance of the subcutaneous tumors in mice treated with HCT-15 cells (1 × 10^7^) or HCT-15/FU cells (1 × 10^7^) in the presence or absence of 1ng /ml TGFβ1. **B and C.** Weight and volume of the subcutaneous tumors **(A)**. **D.** Microvessel density (MVD) of the subcutaneous tumors **(A)** was assessed by CD34 immunohistochemical staining. Scale bars, 100 μm.

Finally, we explored the proliferation and angiogenesis of HCT-15 and HCT-15/FU cells in vivo, and the role of TGFβ1 in these processes. HCT-15 and HCT-15/FU cells were injected into nude mice, followed by the injection of 1.27 mM 5-FU respectively every other day. In addition to 5-FU, the HCT-15/FU group was treated with or without 1 ng/ml TGFβ1. As shown in Figure 5, the tumor volumes, weight and microvessel density (MVD) in the HCT-15/FU group were distinctly higher than those in the HCT-15 group, and the addition of TGFβ1 counteracted this tendency. These data demonstrated that under treatment with 5-FU, HCT-15/FU cells showed more vigorous proliferation and angiogenic capacity than HCT-15 cells in vivo, and activation of the Smad signaling pathway could partly reverse this tendency.

## Discussion

Resistance to chemotherapy is one of the significant obstacles in the clinical treatment of cancer. It is well recognized that tumor cells can modify their microenvironment, including by increasing angiogenesis. However, it remains unknown how chemoresistant cancer cells regulate angiogenesis. In this study, we found that the CM and exosomes derived from HCT-15/FU cells promoted angiogenesis more strongly than those from HCT-15 cells. Our results indicated that HCT-15/FU exosomes contain high levels of GDF15, and enhance angiogenesis by inhibiting the Smad signaling pathway and increasing the expression of POSTN. The application of TGFβ1 can partially reverse this trend and reduce tumor proliferation and MVD in vivo.

Increasing evidence suggests that exosomes transfer genetic information between cells, thereby causing changes in the genes and phenotypes of recipient cells 20-23. In this study we isolated exosomes from HCT-15 and HCT-15/FU CMs and verified that these exosomes could be incorporated into HUVECs. Compared with the control and HCT-15 exosomes, HUVECs cultured with HCT-15/FU exosomes exhibited increased proliferation, migration and tube formation. The numbers of microvessels surrounding rat abdominal aorta were also increased. These results indicate that the exosomes of drug-resistant cells can increase the angiogenic capacity of endothelial cells. The identification of the specific biomolecules in the exosomes of drug-resistant cells responsible for these changes, and the corresponding genetic changes in endothelial cells, will be of considerable theoretical and practical importance.

Evidence indicates that GDF-15 plays different roles in different stages of tumor progression, by acting on the Smad signaling pathway. Although functional studies of its roles in colorectal cancer are limited and controversial, GDF-15 associated functions are not invariable but pleiotropic 14, 24, 25. The effects of intracellular GDF15 and extracellular GDF15 are also different, or even opposite, depending upon the composition of the microenvironment 14, 26.

In our study, we found that the GDF15 was highly expressed in HCT-15/FU exosomes, but expressed at low levels in HCT-15/FU cells. Our research provided evidence that GDF15 may act as a tumor suppressor in cancer cells, and low expression of the protein contributes to the development of 5-FU resistance in colon cancer. Continuous 5-FU treatment caused drug-resistant cells to transfer GDF-15 proteins to the extracellular matrix. Conversely, secreted GDF-15 could act as a tumor promoter by facilitating angiogenesis in the tumor microenvironment. However, the way in which extracellular stress, such as chemotherapy, regulates the intracellular transcription and translation of GDF15 and its secretion into the microenvironment is still not fully understood, and future research should focus on this area.

Among the exosomal GDF15-mediated effects which contribute to increased angiogenesis are TGF-βⅢ receptor activation and Smad signaling pathway inhibition. Previous research suggests that nuclear GDF15 inhibits the Smad pathway by interrupting the Smad complex 27. After the exosomes are internalized by HUVEC cells, the specific localization of GDF15 in cells, and the complementary mechanism of inhibiting the Smad signaling pathway need further study.

Evidence to date indicates that POSTN, an extracellular matrix protein, is overexpressed in many types of human tumors, and can promote angiogenesis by enhancing the pro-angiogenic potency of endothelial cells 28, 29. Our studies found that up-regulation of GDF15 expression in HUVECs was accompanied by increased expression of POSTN, indicating that GDF15 secreted by tumor cells modulates POSTN levels in the extracellular matrix. Intraperitoneal perfusion therapy targeting the GDF15-POSTN axis may reduce intraperitoneal metastasis and the formation of ascites in patients with colon cancer, but this approach needs to be further validated in vivo and in clinical trials. In our study, we further verified the effect of exosomal GDF15 by activating the downstream signaling pathway, more convincing data identifying specific inhibitors of exosomal GDF15 or blocking of exosomes must be obtained, to increase our understanding of the relevant mechanisms involved.

## Conclusion

In summary, our findings demonstrated that GDF15-enriched exosomes from HCT-15/FU cells could suppress the Smad signaling pathway and increase the level of POSTN in HUVECs, consequently facilitate angiogenesis. Our results also supported the potential of the GDF15-POSTN axis as a novel target for the development of new anti-angiogenic therapies in colon cancer.

## Supplementary Material

Supplementary figures and table.

## Abbreviations

5-FU: 5-fluorouracil
GDF15: growth/differentiation factor 15
VEGF: vascular endothelial growth factor
CM: conditioned medium
POSTN: periostin
HUVECs: human umbilical vein endothelial cells
qRT-PCR: quantitative reverse transcription PCR
MVD: microvessel density
