# Supplementary material for: Exosomes derived from 5-fluorouracil-resistant colon cancer cells are enriched in GDF15 and can promote angiogenesis
Source: J Cancer. 2020 Oct 18;11(24):7116–26. doi: 10.7150/jca.49224 (PMC7646166; doi:10.7150/jca.49224)

Supplement Table

Table S1. Information of the qRT-PCR primer sequences.

Supplement Figures

Figure S1. IC_{50} of 5-FU for human colon cancer HCT-15 and HCT-15/FU cells.

Figure S2. Proliferation assay of HUVECs treated with various concentrations of HCT-15 and HCT-15/FU exosomes by live-cell imaging experiment.

Table S1

Primer Name	5′ to 3′
DPP4 (F)	GGGTCACATGGTCACCAGAG
DPP4 (R)	TCTGCGTCGTTAAATTGGGCATA
FSCN (F)	CCAGGGTATGGACCTGTCTG
FSCN (R)	GTGTGGGTACGGAAGGCAC
GDF15 (F)	GACCCTCAGAGTTGCACTCC
GDF15 (R)	GCCTGGTTAGCAGGTCCTC
GSTK1 (F)	TCTGGAAAAGATCGCAACGC
GSTK1 (R)	GCCCAAAGGCTCCGAATCTG
SPTA (F)	GCCAACTCAGGAGCCATTGTT
SPTA (R)	CGGGTCCGTATGGTTTCAGAT
TXNRD1 (F)	ATATGGCAAGAAGGTGATGGTCC
TXNRD1 (R)	GGGCTTGTCCTAACAAAGCTG
AKR1C1 (F)	TTCATGCCTGTCCTGGGATTT
AKR1C1 (R)	CTGGCTTTACAGACACTGGAAAA
PFKP (F)	GCATGGGTATCTACGTGGGG
PFKP (R)	CTCTGCGATGTTTGAGCCTC
SQSTM1 (F)	GCACCCCAATGTGATCTGC
SQSTM1 (R)	CGCTACACAAGTCGTAGTCTGG
GFPT1 (F)	AACTACCATGTTCCTCGAACGA
GFPT1 (R)	CTCCATCAAATCCCACACCAG
GAPDH (F)	GGAGCGAGATCCCTCCAAAAT
GAPDH (R)	GGCTGTTGTCATACTTCTCATGG
TGFBR1 (F)	GCTGTATTGCAGACTTAGGACTG
TGFBR1 (R)	TTTTTGTTCCCACTCTGTGGTT
TGFBR2 (F)	AAGATGACCGCTCTGACATCA
TGFBR2 (R)	CTTATAGACCTCAGCAAAGCGAC
TGFBR3 (F)	TGGGGTCTCCAGACTGTTTTT
TGFBR3 (R)	CTGCTCCATACTCTTTTCGGG

Figure S1

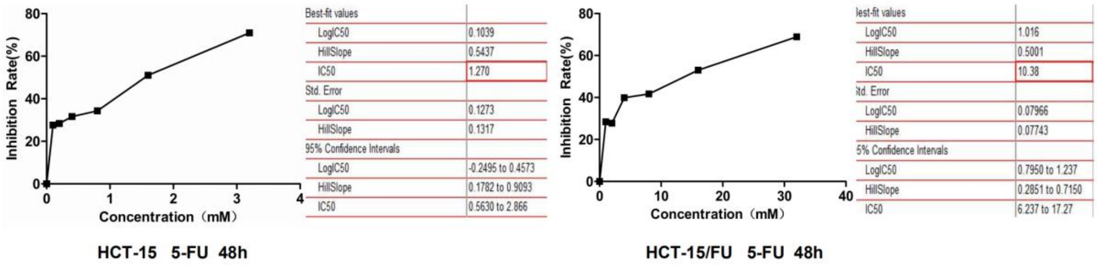

Figure S2

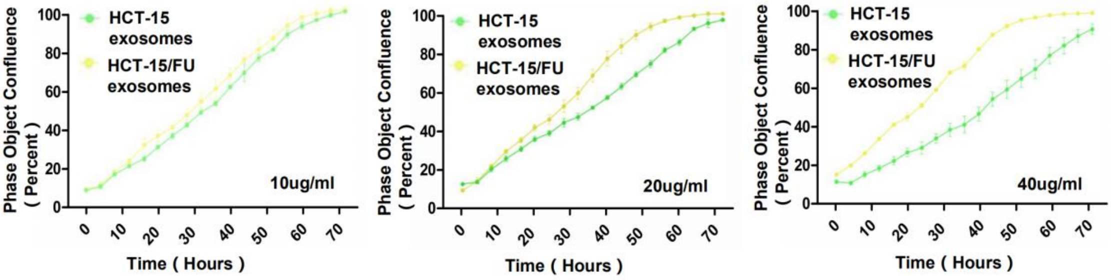

Supplement: Supplementary file 1 — Supplementary figures and table. [file jcav11p7116s1.pdf]
